# Modeling cancer-associated hypercoagulability using glioblastoma spheroids in microfluidic chips

**DOI:** 10.1016/j.rpth.2024.102475

**Published:** 2024-06-17

**Authors:** Maaike Y. Kapteijn, Monika Yanovska, El Houari Laghmani, Rudmer J. Postma, Vincent van Duinen, Betül Ünlü, Karla Queiroz, Anton Jan van Zonneveld, Henri H. Versteeg, Araci M.R. Rondon

**Affiliations:** 1 Division of Thrombosis and Hemostasis, Einthoven Laboratory for Vascular and Regenerative Medicine, Leiden University Medical Center, Leiden, the Netherlands; 2 Division of Nephrology, Einthoven Laboratory for Vascular and Regenerative Medicine, Leiden University Medical Center, Leiden, the Netherlands; 3 Mimetas BV, Oegstgeest, the Netherlands

**Keywords:** anticoagulants, cancer-associated thrombosis, extracellular vesicles, glioblastoma, organ-on-a-chip

## Abstract

**Background:**

Cancer increases the risk of venous thromboembolism, and glioblastoma is one of the cancer types with the highest risk of venous thromboembolism (10%-30%). Tumor-intrinsic features are believed to affect vascular permeability and hypercoagulability, but novel models are required to study the pathophysiological dynamics underlying cancer-associated thrombosis at the molecular level.

**Objectives:**

We have developed a novel cancer-on-a-chip model to examine the effects of glioblastoma cells on the deregulation of blood coagulation.

**Methods:**

This was accomplished by coculturing vessel-forming human umbilical vein endothelial cells with glioblastoma spheroids overexpressing tissue factor (TF), the initiator of coagulation (U251 lentivirus, LV-TF) or an LV-control (U251 LV-Ctrl) in an OrganoPlate Graft platform.

**Results:**

Using a modified thrombin generation assay inside the cancer-on-a-chip, we found that U251 LV-Ctrl and U251 LV-TF spheroids promoted an increased procoagulant state in plasma, as was shown by a 3.1- and 7.0-fold increase in endogenous thrombin potential, respectively. Furthermore, the anticoagulant drug rivaroxaban and TF coagulation-blocking antibody 5G9 inhibited the activation of blood coagulation in U251 LV-TF spheroid-containing graft plates, as was shown by a reduced endogenous thrombin potential (4.0- and 4.4-fold, respectively).

**Conclusion:**

With this study, we present a novel 3-dimensional cancer-on-a-chip model that has the potential to be used in the discovery of new anticoagulant drugs and identification of optimal anticoagulant strategies for glioblastoma and other cancer types.

## Essentials

• Thrombosis leads to increased mortality in cancer patients.
• Cancer hypercoagulability-on-a-chip is a coculture of a cancer spheroid and human umbilical vein endothelial cell–based vessels.
• Thrombin generation inside the chip can be inhibited by anticoagulant modalities.
• This novel model can be used for different cancer types and other pathologies.

## 1 Introduction

Cancer is a well-known risk factor for venous thromboembolism (VTE). The presence of a malignancy increases the risk of VTE 9-fold [1], and cancer-associated thrombosis (CAT) results in a less favorable prognosis in comparison with cancer or VTE alone [2,3]. Importantly, thromboembolism is the second leading cause of death after cancer itself [4]. The transmembrane glycoprotein tissue factor (TF) is often presented as the link between cancer and thrombosis, being the initiator of coagulation, and is overexpressed in virtually all cancer types [5].

Mouse models have been essential for our understanding of (cancer-associated) thrombogenesis. However, they are not ideal since spontaneous VTE does not occur in animals, and hemodynamics differ significantly between species [6]. Therefore, innovative 3-dimensional (3D) humanized cell culture models to investigate the dynamic interaction between the tumor and the coagulation system are required to get more insight into the underlying pathophysiological mechanisms of CAT.

Glioblastoma is the most aggressive type of primary brain cancer and exhibits a 10% to 30% VTE prevalence during the course of the disease, which is one of the highest VTE incidences among cancers [7,8]. The median expected survival for glioblastoma patients is only 15 months despite extensive treatment, consisting of tumor resection, radiotherapy, and chemotherapy [9]. VTE is often observed within the first 6 months after glioblastoma diagnosis but remains high throughout the whole disease trajectory [8]. Thromboprophylaxis is not generally prescribed due to the increased risk of intracranial hemorrhage [10]. The highly increased risk of VTE in glioblastoma patients suggests a role for tumor-intrinsic features that influence hypercoagulability, such as tumor-expressed genes that drive procoagulant factors [11,12], but more research is warranted to decipher the dynamics behind glioblastoma-related VTE.

Organ-on-a-chip is one of the novel 3D cell culture models that is suitable for investigating the dynamic interactions between tumor cells and their microenvironment due to its opportunity to incorporate several cell types in a 3D scaffold together with microfluidics on a standardized perfusable cell culture platform [13]. Although microfluidic models for cancer and the nervous system have been extensively developed, no model is currently available that allows for the direct evaluation of prothrombotic effects of tumor cells. Here, by combining the presence of a glioblastoma spheroid with the formation of human umbilical vein endothelial cell (HUVEC)-based vessels, all-in-1 platform but separated by a glass and extracellular matrix, we have developed a cancer-on-a-chip system that can be used to examine the communication between glioblastoma cells and the vasculature. This proof-of-principle animal-free model provides novel opportunities for studying the 3D tumor microenvironment *in vitro* and will contribute to our knowledge of CAT.

## 2 Methods

### 2.1 Cell culture

Primary HUVECs were isolated from newborn’s umbilical cords obtained from the Department of Obstetrics at the Leiden University Medical Center, as previously described [14]. They were cultured in endothelial cell growth medium 2 (EGM2, C-22011, PromoCell) containing supplement mix (C39216, PromoCell) and used until the third passage.

The wild-type human glioblastoma cell line U251 was kindly provided by Prof Dr Janusz Rak (McGill University, Canada). U251 cells were cultured in Dulbecco’s modified eagle medium (DMEM, 1966-029, Gibco), supplemented with 10% fetal bovine serum, (Bodinco), 1% L-glutamine (G7513, Sigma-Aldrich), and 1% penicillin-streptomycin (15070-063100x, Gibco). All cells were incubated in a humidified incubator at 37 °C and 5% CO_2_.

Spheroids of U251 cells were formed using round-bottom low-attachment 96-well plates (7007, Corning). For each spheroid, 10,000 cells were seeded in 200 μL of supplemented DMEM. After 4 days in culture, the spheroids were formed and used for experiments.

### 2.2 U251 lentiviral transduction and knockdown

Lentivirus (LV) based on the pLV.CMV.bc.PURO vector (control [Ctrl], Sigma-Aldrich) or the pLV.CMV.bc.PURO vector containing human TF complementary DNA (incorporated via *PmeI/EcoRV*, Sigma-Aldrich) in combination with the LV packaging plasmids pVSV-G and psPAX2 was produced using human embryonic kidney 293 expressing the SV40 large T antigen cells and polyethylenimine (Polysciences). Knockdown variants short hairpin RNA TF 3494 or short hairpin 0001Ctrl (Mission Library; Sigma-Aldrich) were also generated. U251 cells were plated in a 6-well plate at 30% confluency. After 24 hours, cells were LV transduced with 100 μL (overexpression) or 700 μL (knockdown) of virus particles and 6 to 8 μg/mL polybrene per well. Twenty-four hours after LV transduction, the medium was refreshed, and puromycin (A1138-03, Gibco) was added for cell selection (1-2 μg/mL per well). All generated cell lines were validated using western blotting.

### 2.3 Extracellular vesicles

To generate extracellular vesicles (EVs), 1 × 10^7^ U251 cells or HUVECs were seeded in four 175 cm^2^ flasks and cultured for 24 hours. Afterward, cells were cultured in serum-free DMEM or EGM for 24 hours. The medium was collected and centrifuged at 1000 × *g* for 10 minutes to remove cell debris, followed by 2 centrifugations (without break) at 20,000 × *g* for 1 hour using polycarbonate bottles (#355618, Beckman Coulter) in a 50.2 Ti rotor and an Optima XE-90 ultracentrifuge (Beckman Coulter) [15,16]. For the second centrifugation, phosphate-buffered saline (PBS) was added to the pellet. Protein quantification was performed using DC Protein Assay (5000111, Bio-Rad).

### 2.4 Microfluidic culture OrganoPlate Graft

Rat tail collagen type I (5 mg/mL, 3447-020-01, R&D Systems) was mixed with 10% 37 g/L Na_2_CO_3_ (MC1063925000, Merck) and 10% 1 M HEPES buffer (H3375, Sigma-Aldrich) on ice. Collagen mix (2.4 μL) was dispensed into the gel inlet of each chip of an OrganoPlate Graft (6401-400-B, Mimetas) and incubated for 15 minutes at 37 °C and 5% CO_2_. Next, 2 μL of 1 × 10^7^ HUVECs/mL was added to the medium inlets, followed by 50 μL of EGM2, and incubated for 2 hours at 37 °C and 5% CO_2_. Afterward, 50 μL of EGM2 was added to the medium outlet wells and graft chamber. The plate was placed on an interval rocker platform (Mimetas) at 7° inclination with cycle time of 8 minutes, allowing a continuous bidirectional flow for at least 4 days. The previously generated spheroids were washed twice with PBS and kept on EGM2. After 1 hour, U251 spheroids were added to the graft chambers using a micropipette. The coculture of HUVEC vessels and 1 spheroid/chip was incubated for 4 days on the rocker platform.

### 2.5 Immunostaining, imaging, and quantification

HUVEC-based vessels and U251 spheroids were fixed with 4% paraformaldehyde for 10 minutes and washed with Hank’s balanced salt solution containing calcium and magnesium (HBSS+, 14025-050, Gibco). Permeabilization and blocking buffer containing 0.5% glycine (56-40-6, Merck), 0.8% Triton X-100 (Art11869, Merck), and 2% bovine serum albumin (BSA, A7030, Sigma-Aldrich) in HBSS+ was added for 2 hours. Primary antibodies (Table) were diluted in 0.3% Triton X-100 and 3% BSA and kept overnight at 4 °C. The cells were washed with 0.3% Triton X-100 in HBSS+. Secondary antibodies (Table) were diluted in 0.05% glycine, 0.3% Triton X-100, and 2% BSA in HBSS+ and kept for 2 hours at room temperature. Hoechst 33342 diluted in PBS was incubated for 30 minutes at room temperature.

**Table:** Antibodies, probes, and dyes used for immunostaining.

Antibodies, probes, and dyes	Dilution	Catalog No. and supplier
Mouse antihuman VE-cadherin	1:150	555661, BD Biosciences, United States
Mouse antihuman ICAM-1	1:100	Clone BBI-I1 (11C81), BBA3, R&D, United States
Rabbit antihuman VWF	1:1000	A0082, Dako, United States
Goat antimouse IgG Alexa 488	1:250	A1100, Invitrogen, United States
Goat antirabbit IgG Alexa 647	1:250	A21244, Invitrogen, United States
Phalloidin rhodamine	1:250	R415, Invitrogen, United States
Hoechst 33342	1:2000	H-3569, Molecular Probes, United States

ICAM-1, intercellular adhesion molecule 1; IgG, immunoglobulin G; VE, vascular endothelial; VWF, von Willebrand factor.

The plate was imaged using an automated confocal microscope (ImageXpress Micro confocal microscope, Molecular Devices), using the 4′,6-diamidino-2-phenylindole, fluorescein isothiocyanate, tetramethylrhodamine (TRITC), and cyanine-5 filter/mirror sets. For 3D cross sections, z-series of 2 μm step-size and 500 μm total z-height were acquired using a Nikon plan APO lambda 20×, with numerical aperture of 0.75 objective lens. 3D reconstruction was performed using Imaris (Oxford Instruments). To quantify vascular endothelial (VE)-cadherin, max projections were created of a z-series 0.5 μm step-size and 10 μm of total z-height, imaged using a Nikon plan APO lambda 40×, with numerical aperture of 0.95 objective lens. A detailed description of all immunofluorescence quantification can be found in the Supplementary Methods.

### 2.6 Permeability assay

A fluorescent solution of 155 kDa TRITC-dextran (T1287, Sigma-Aldrich) was made using prewarmed EGM2 (0.5 mg/mL). Prewarmed EGM2 (20 μL) was added to the graft, gel inlet, and right medium inlet and outlet of each chip. Subsequently, 40 μL and 20 μL of the TRITC-dextran solutions were added to the left medium inlet and outlet per chip, respectively. The plate was imaged immediately with an ImageXpress Micro confocal system at 1-minute intervals for 20 minutes in total.

The fluorescent intensity of the left medium channel (representing the endothelial microvessel) and the adjacent gel channel were quantified using FIJI [17]. Regions of interest of the same size were defined for quantification. The apparent permeability coefficient (Papp) was calculated as previously described [18] to determine vessel permeability.

### 2.7 Thrombin generation

For this study, the calibrated automatic thrombography method developed by Hemker et al. [19] was adapted to quantify plasma-derived thrombin generation within the HUVEC-based vessels by monitoring the cleavage of a fluorogenic substrate. The chips were washed 2 times with 50 μL of prewarmed HBSS without calcium or magnesium (HBSS−, 14175-53, Gibco). Prewarmed HBSS− (40 μL) was added to the graft gel chamber, gel inlet, and right medium inlet and outlet of each chip. Normal pooled plasma (Cryopep) was diluted in HBSS− in a 1:1 ratio with 50 μg/mL of corn trypsin inhibitor (Haematologic Technologies) to inhibit contact activation. Subsequently, 40 μL of the plasma solution was added to the left medium inlet and outlet (80 μL in total). For the initiation of thrombin formation, a multichannel pipette was used to manually homogenize and add 10 μL of a prewarmed fluorescence-substrate buffer containing calcium chloride (FluCa-Kit, TS50.00, Thrombinoscope) to the wells previously filled with plasma, resulting in a final reaction volume of 100 μL per chip (20 μL FluCa-Kit and 80 μL diluted plasma). The plate was imaged immediately with an automatic ImageXpress Micro confocal microscope, set to obtain an image of all chips every minute for 1 hour. The detected fluorescence in the left medium channel (representing the endothelial microvessel) was quantified using FIJI [17]. For the quantification of the obtained images, regions of interest of the same size were defined.

Prior to adding the fluorescence-substrate buffer, 2 μL of thrombin calibrator 730 nm (TS20.00, Thrombinoscope) was added to some chips to quantify thrombin generation in plasma. Other chips seeded with endothelial cells only (no U251 spheroids) were used as negative Ctrl. Furthermore, selected chips were incubated with 10 ng/mL tumor necrosis factor alfa (TNF-α, H8916, Sigma-Aldrich) at 37 °C and 5% CO_2_ for 4 hours as a positive Ctrl.

### 2.8 Anticoagulant blocking experiments

Fifteen minutes prior to the experiment, anticoagulant compounds were diluted in a solution of plasma, corn trypsin inhibitor, and HBSS− to a final concentration of 50 μg/mL anti-TF 5G9, 50 μg/mL TIB115, or 100 ng/mL rivaroxaban (C2138, Alsachim) at 37 °C. Anti-TF 5G9 and TIB115 were kindly provided by Prof Dr Wolfram Ruf, Institute of Molecular Biology, DE, Germany.

### 2.9 Western blot

A total of 5 × 10^5^ cells/well were seeded in 6-well plates and harvested as described previously [16]. Cells and EVs were lysed in Tris-Glycine SDS sample buffer 2× (LC2676, Novex, Thermo Fisher). Anti-TF mouse TF9-10H10, also kindly provided by Prof Dr Ruf, and anti-GAPDH rabbit (D16H11, Cell Signaling), were used as primary antibodies, and goat antirabbit or antimouse IgG HRP (ab97051, ab97023 Abcam) were used as secondary antibodies.

### 2.10 Statistical analysis

Experiments were conducted a minimum of 3 times with at least 3 replicates per group unless specified otherwise. Outliers were removed when exceeding 1.5 IQR above or below the upper or lower quartile, respectively. Results were analyzed using unpaired Student’s *t*-test or analysis of variance plus Dunnett’s multiple comparison test with GraphPad Prism 9.3.1 (GraphPad Software). Significant differences compared with Ctrl are indicated as follows: *P* < .10 (#), *P* < .05 (∗), *P* < .01 (∗∗), *P* < .001 (∗∗∗), and *P* < .0001 (∗∗∗∗).

## 3 Results

### 3.1 Coculture of glioblastoma spheroids and HUVECs inside the organ-on-a-chip

**Figure 1:**
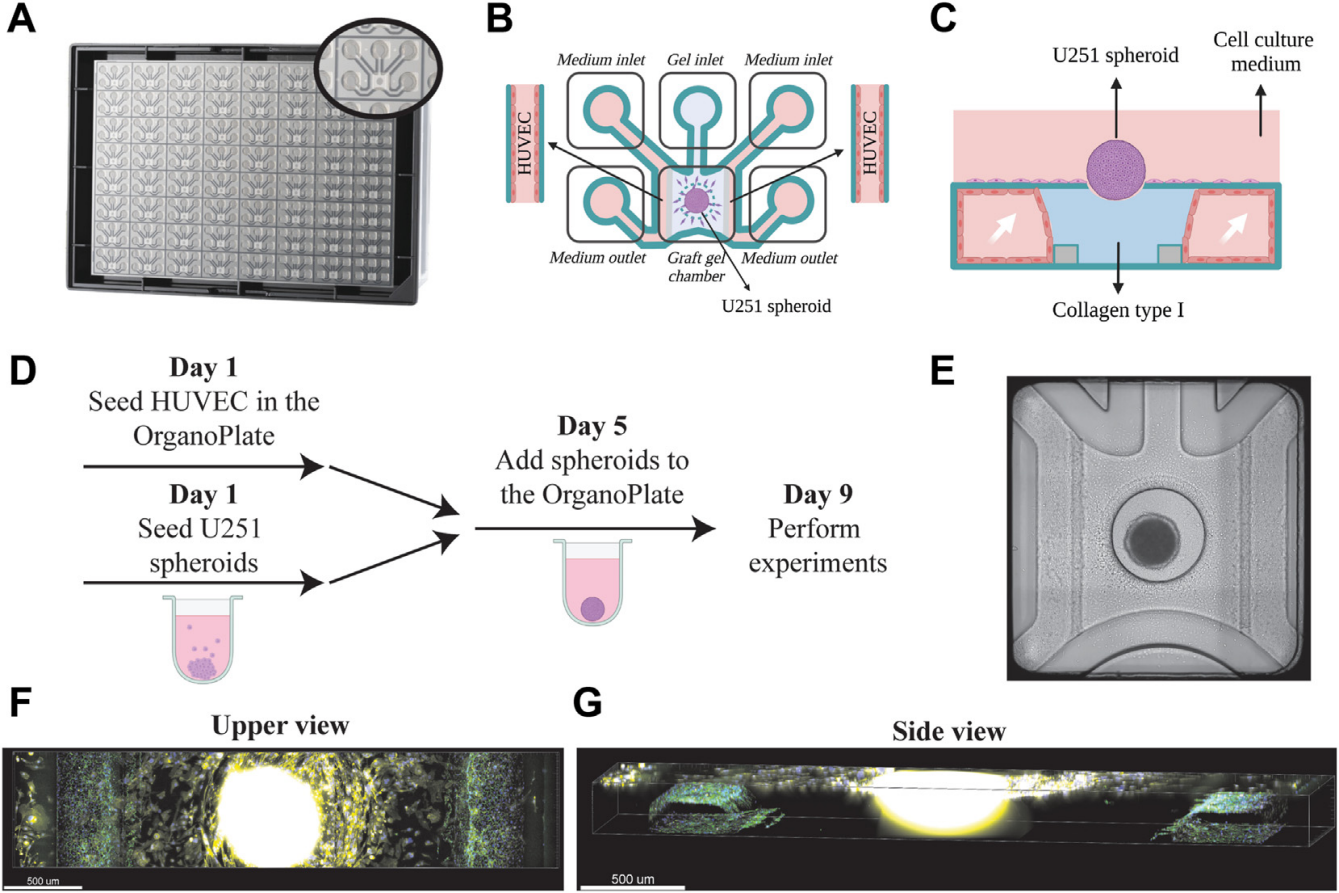
Cancer-associated hypercoagulability-on-a-chip for glioblastoma. (A) Bottom view of an OrganoPlate Graft consisting of 64 independent perfusable tissue culture chips. (B) Schematic upper view representation of one OrganoPlate Graft tissue culture chip. (C) Side view of a chip showing growth of human umbilical vein endothelial cell (HUVEC) vessels in cell culture medium next to the extracellular matrix, consisting of collagen type I. The white arrows represent the bidirectional flow. (D) Schematic depiction of the experimental setup. (E) Example of a live coculture of HUVEC vessels and a U251 spheroid. (F, G) Immunofluorescence of the upper view (F, bar = 500 μm) and side view (G, bar = 500 μm) of the middle section of a chip (blue, DNA/Hoechst 33342; green: vascular endothelial-cadherin; yellow: phalloidin).

**Supplementary Figure 1:**
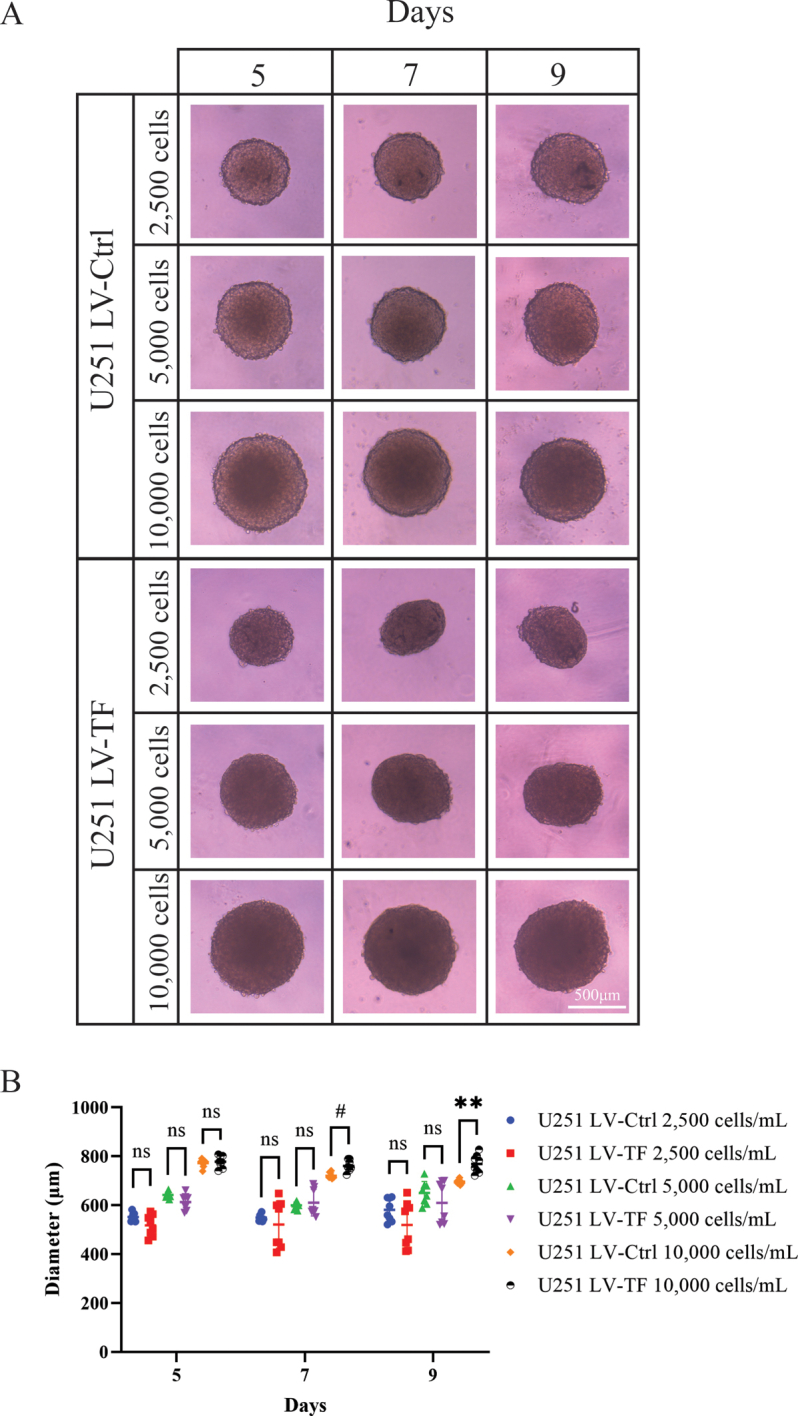


**Supplementary Figure 2:**
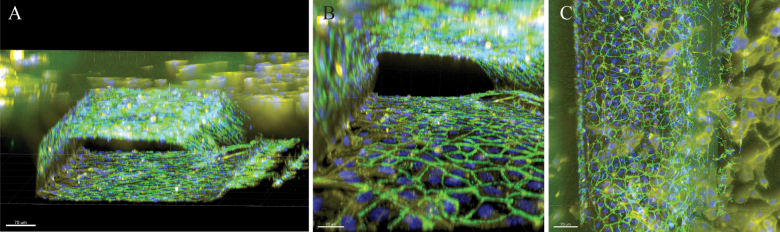


To develop a cancer-on-a-chip model for cancer-related hypercoagulability, we used the Mimetas OrganoPlate Graft platform consisting of 64 individual chips with an open chamber in the middle connected to 2 perfusable vessels (Figure 1A–C). In brief, after addition and solidification of collagen type I in the gel inlet, HUVECs were seeded in the perfusion channels, where the vessels were formed in direct lateral contact with collagen type I (Figure 1B, C). Simultaneously, U251 spheroids were generated by seeding 10,000 cells/well in round-bottom low-attachment 96-well plates. After 96 hours in culture, U251 spheroids (Supplementary Figure S1) were placed in the observation windows of the cancer-on-a-chip (Figure 1D, E) in direct contact with the collagen. The coculture was kept under bidirectional flow for 72 hours until further experiments were performed. Staining of VE-cadherin and phalloidin, as indicated in Figure 1F, G, Supplementary Figure S2, and Supplementary Video, demonstrated proper vessel formation. As shown in Figure 1F, G, some U251 cells were derived from the tumor spheroid migrated on top of the glass but not into the gel toward the endothelial vessels, demonstrating that the tumor cells were physically separated from the vessels.

### 3.2 Effects of glioblastoma spheroids on endothelial barrier function

**Figure 2:**
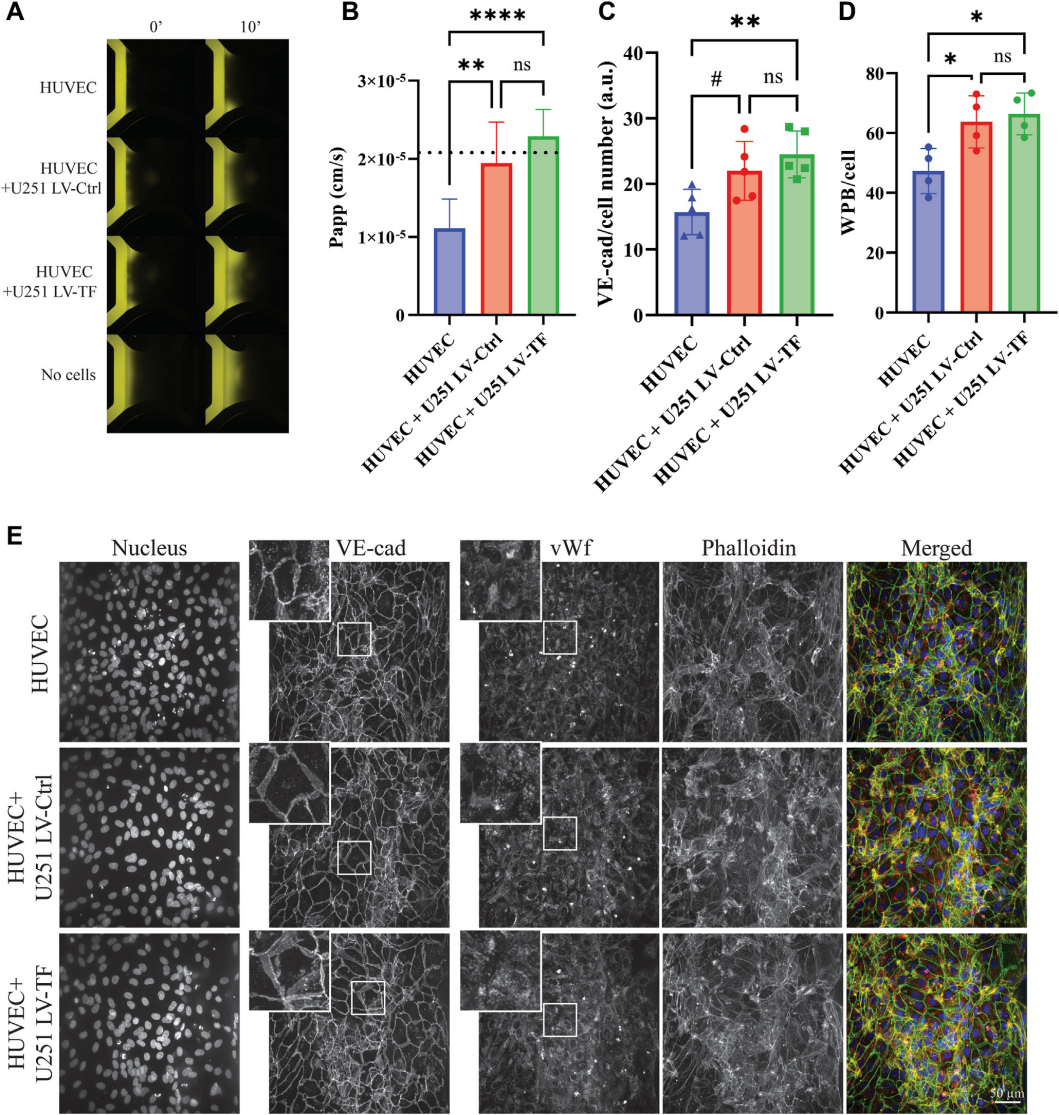
Effects of glioblastoma spheroids on endothelial barrier function. (A) Tetramethylrhodamine-dextran (155 kDa size) was added to human umbilical vein endothelial cell (HUVEC) vessels in the organ-on-a-chip, and vessel permeability was evaluated by imaging fluorochrome diffusion (B). The apparent permeability coefficient (Papp) was calculated to determine the leakiness of the endothelial barrier [18]. The dotted line represents the cell-free condition, which is used as a positive control as there is no endothelial barrier. Representative graphic from one experiment (*n* = 4). (C) Quantification of fluorescence intensity of vascular endothelial (VE)-cadherin (C, *n* = 5). (D) Quantification of the number of Weibel-Palade bodies (WPB) per cell (J, *n* = 4) on HUVEC vessels inside the cancer-on-a-chip in accordance with the protocol described by [20]. Note that the HUVEC-only condition shows less von Willebrand factor (VWF) staining. (E) Immunofluorescence staining and imaging of DNA/Hoechst 33342 (blue), VE-cadherin (green), VWF (red), phalloidin (yellow), and a merged composition, bar = 50 μm. Insert images (2.5×) are shown for VE-cadherin and VWF staining. #*P* < .10. LV-Ctrl, lentivirus control; LV-TF, lentivirus tissue factor; Ns, not significant.

**Figure 3:**
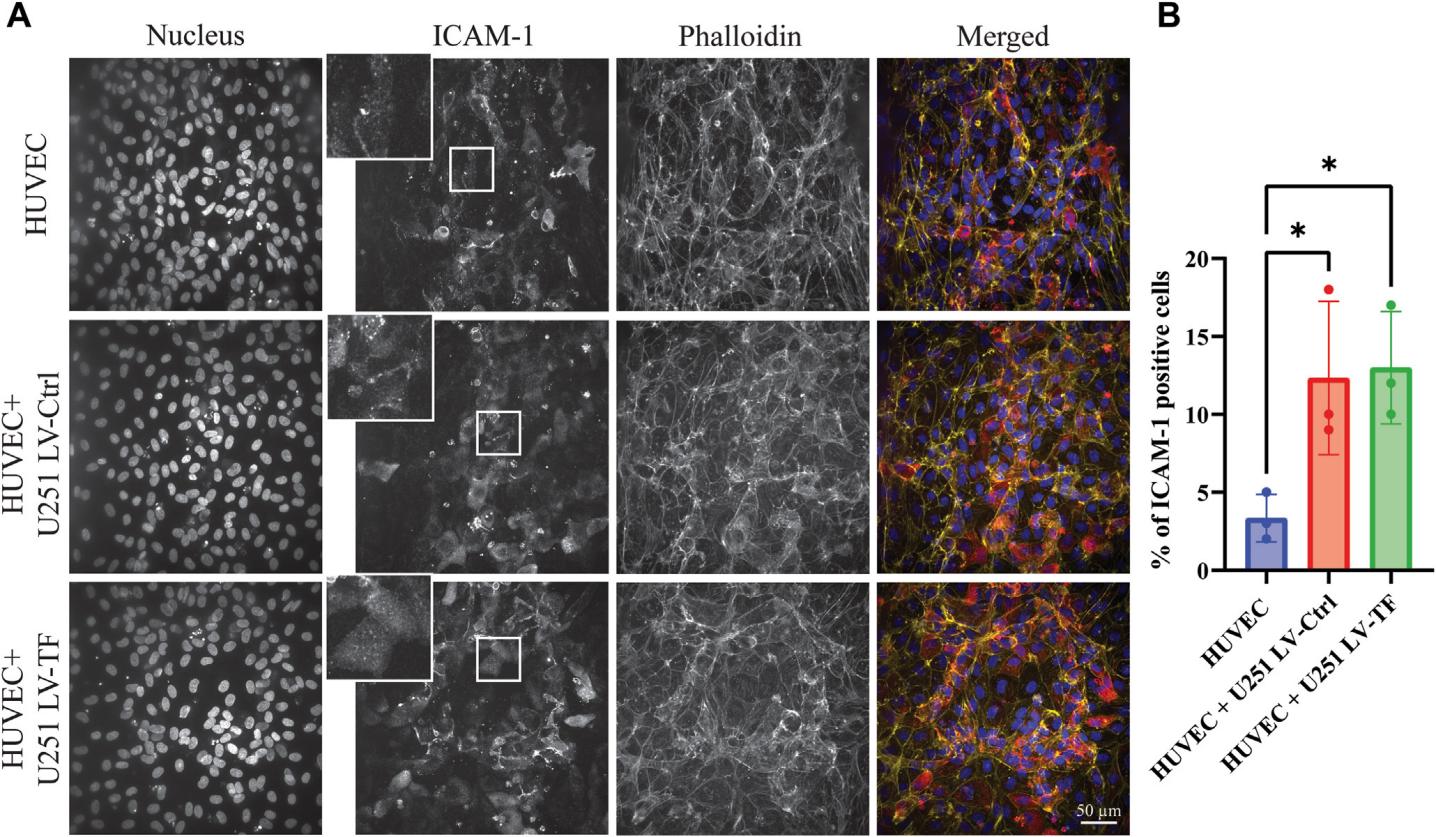
Effects of endothelial activation on intercellular adhesion molecule 1 (ICAM-1) expression. (A) Immunofluorescence staining of human umbilical vein endothelial cell (HUVEC)-based vessels inside the cancer-on-a-chip (DNA/Hoechst 33342 [blue], ICAM-1 [red], phalloidin [yellow], and a merged composition). Bar = 50 μm. (B) Quantification of ICAM-1–positive cells. Hoechst 33342 and phalloidin were used to define the number limits of cells., *n* = 3; ∗*P* < .05. Insert images (2.5×) are shown for ICAM-1. LV-Ctrl, lentivirus control; LV-TF, lentivirus tissue factor.

**Supplementary Figure 3:**
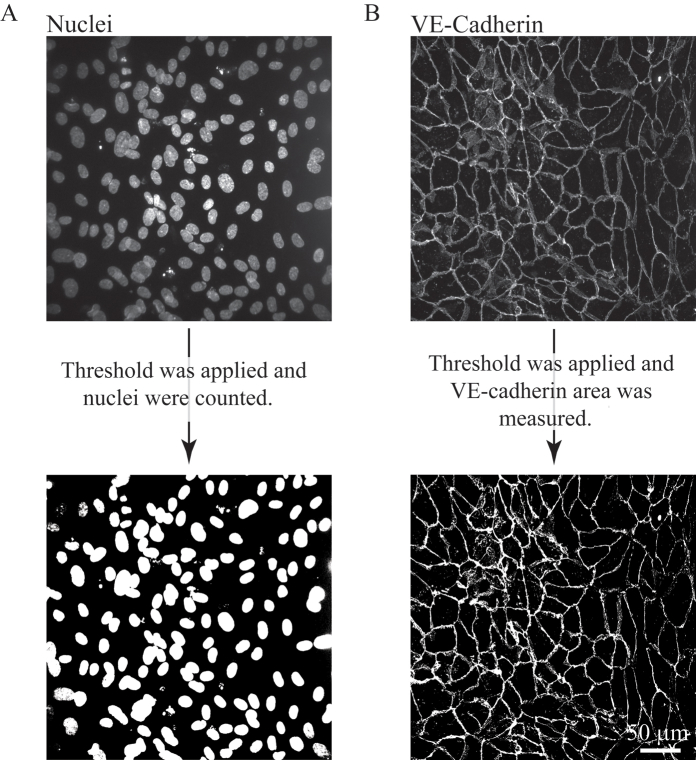


**Supplementary Figure 4:**
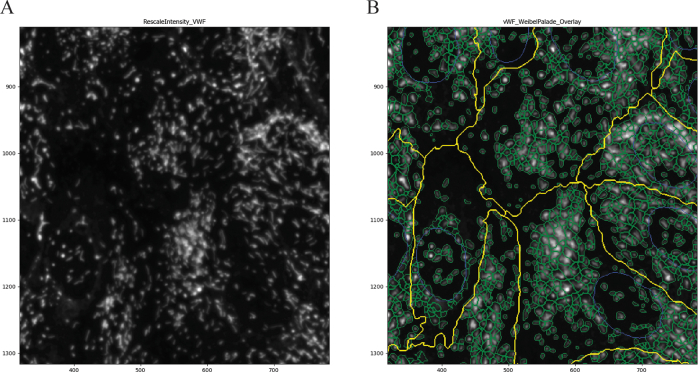


**Supplementary Figure 5:**
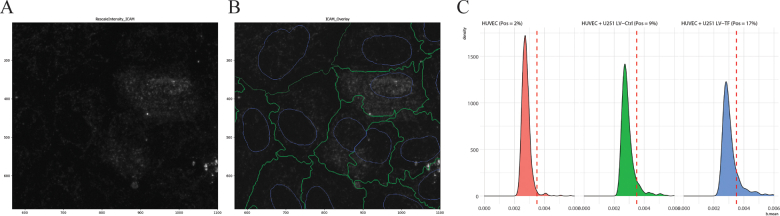


In cancer, vessel integrity is often compromised, resulting in an increased risk of VTE and metastasis. We evaluated the endothelial barrier of HUVECs exposed to U251 spheroids for 72 hours by performing a permeability assay inside the cancer-on-a-chip. The presence of both U251 LV-Ctrl and U251 LV-TF spheroids promoted higher endothelial permeability compared with HUVECs alone (Figure 2A [18]), as represented by the Papp (Figure 2B). Next, immunofluorescence staining of VE-cadherin was used to study vascular integrity. The coculture of HUVECs and U251 LV-TF spheroids promoted more destabilized junctions, as evidenced by a more diffuse VE-cadherin signal in comparison with HUVECs alone, which showed tighter and thinner VE-cadherin staining (Figure 2C, E and Supplementary Figure S3). This is consistent with our Papp results, suggesting increased permeability. We also looked into von Willebrand factor, a glycoprotein involved in both primary hemostasis as well as cancer progression. The number of Weibel-Palade bodies in which von Willebrand factor is stored was increased in the presence of U251 spheroids (Figure 2D, E and Supplementary Figure S4 [20]), demonstrating increased vascular permeability and hypercoagulable potential. Finally, immunofluorescence staining of intercellular adhesion molecule 1 (ICAM-1) showed an increased percentage of ICAM-1 positive cells (Figure 3A, B and Supplementary Figure S5 [21]), suggesting activation of endothelial cells. This could be explained by U251-mediated secretion of growth factors, resulting in communication between the glioblastoma spheroid and HUVECs inside the cancer-on-a-chip.

### 3.3 Thrombin generation inside the cancer-on-a-chip model

**Figure 4:**
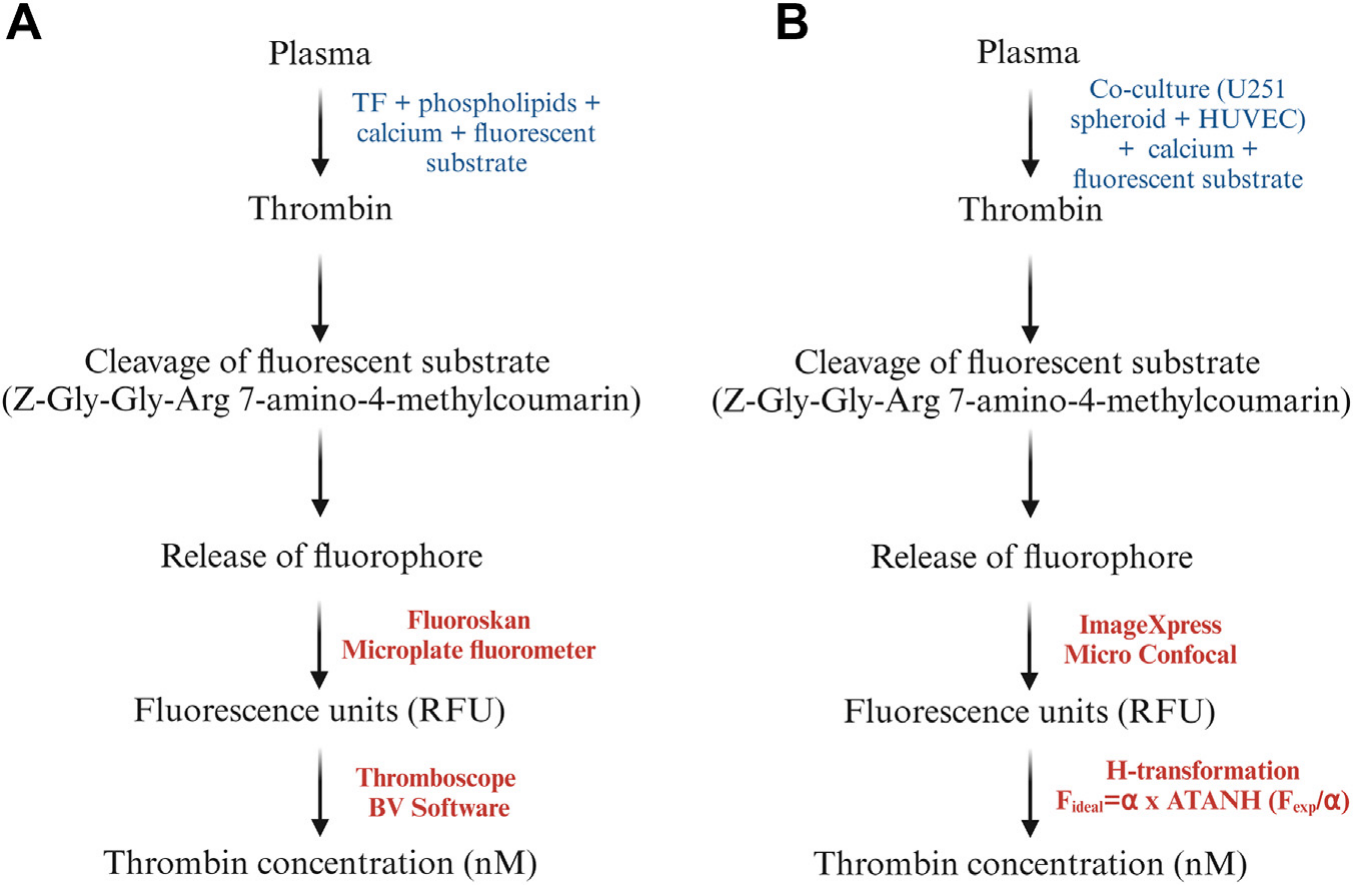
Schematic of the thrombin formation reaction and cleavage of the fluorescent substrate. (A) Calibrated automated thrombogram method on a 2-dimensional 96-well plate developed by Hemker et al. [19]. (B) Adaptation of the method on an OrganoPlate Graft or 2-lane. The figure was adapted from [23]. The ATANH function, also known as arctanh, is the inverse hyperbolic tangent of a number. F, fluorescent intensity; HUVEC, human umbilical vein endothelial cell; RFU, relative fluorescence units; TF, tissue factor.

**Figure 5:**
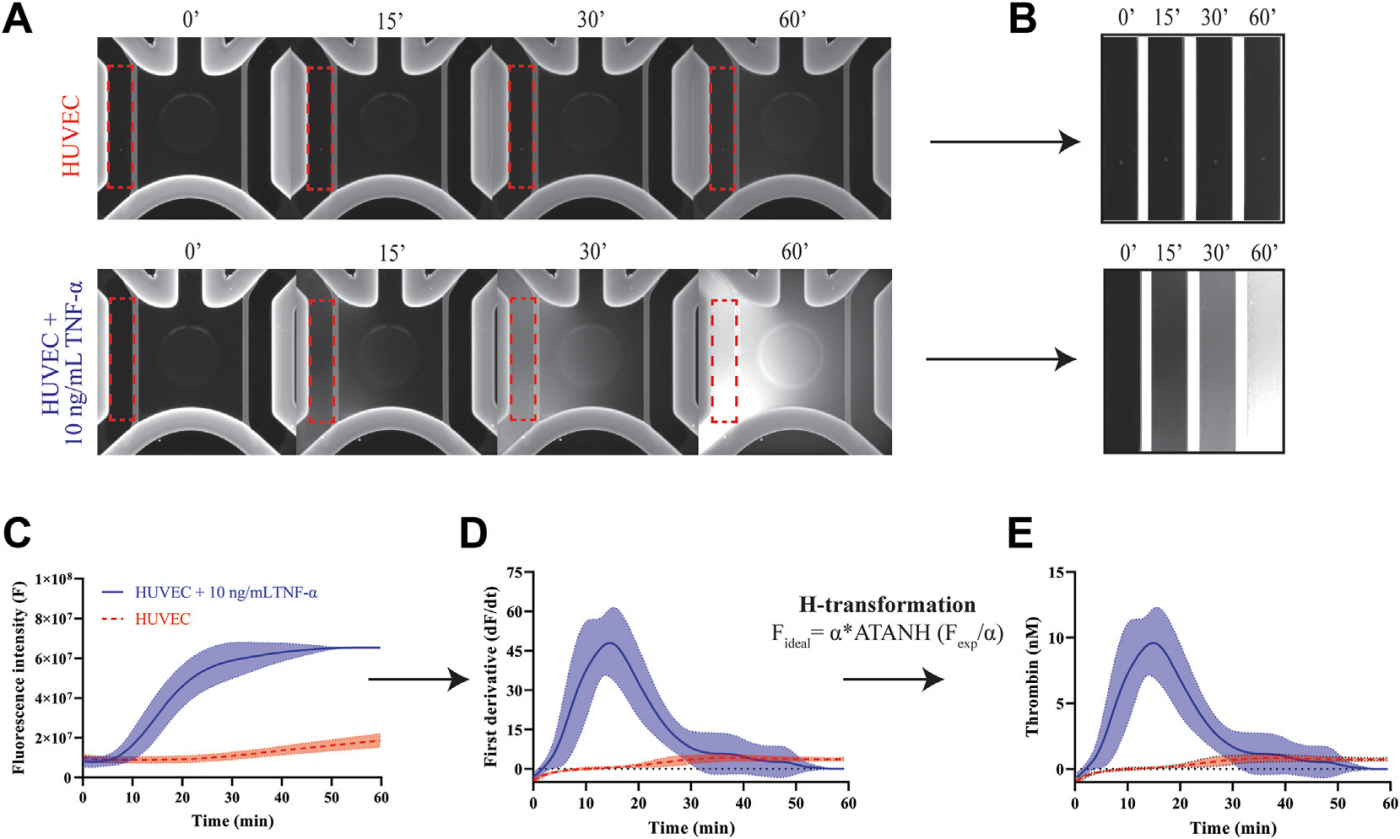
Thrombin generation inside the cancer-on-a-chip model using the adapted calibrated automated thrombinography method by Hemker et al. [19] (A–C) After stimulating human umbilical vein endothelial cell (HUVEC) vessels with tumor necrosis factor alfa (TNF-α) for 4 hours, blood plasma and fluorescence-substrate buffer (containing calcium chloride and a peptide present on the FluCa-Kit) were added to a vessel of the cancer-on-a-chip. The peptide is cleaved by thrombin, liberating a fluorochrome, which is measured over time using an automatic confocal. The fluorescence intensity (F) was quantified using FIJI. (D) A first derivative was calculated from the fluorescence intensity values. (E) The data were transformed with the H-transformation formula. The ATANH function is the inverse hyperbolic tangent of a number. The highlighted area represents the SD. Graphics are representatives of one experiment. Experiments were performed at least 3 times, and each condition was conducted at least in triplicate.

**Supplementary Figure 6:**
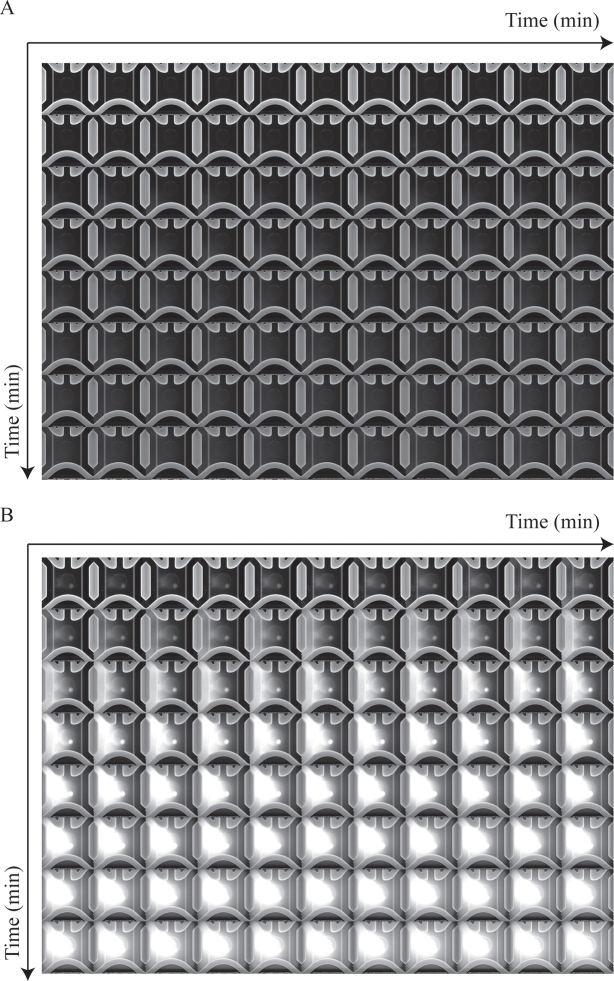


To evaluate activation of the coagulation cascade in the cancer-on-a-chip model, we measured real-time thrombin generation in blood plasma inside the vessels by adapting a model developed by Hemker et al. [19] (see Methods, Figure 4 [19,22]). This method requires the external addition of TF and phospholipids for coagulation initiation in the absence of TF-expressing cells, while our adaptation relies on the presence of endogenous TF, most likely present on spheroid-derived EVs or HUVECs inside the cancer-on-a-chip (as a result of tumor cell-derived proinflammatory cytokines) [19,23]. To validate this model, we stimulated HUVECs on the chip with TNF-α, which is known to promote HUVEC-dependent TF expression [24]. This was followed by perfusion of the vessels with blood plasma and fluorescence-substrate buffer (FluCa-Kit), which showed increased fluorescence over time, demonstrating generation of thrombin in this system (Figure 5A, B and Supplementary Figure S6). To determine the amount of thrombin generation, the fluorescence intensities were converted into their respective thrombin curves using several calculation steps [19,22] (Figure 5C–E). Dedicated software (Thrombinoscope BV Software) [23] could not be used with the OrganoPlate Graft. Therefore, fluorescent intensity (F) of the left channel (representing the perfused endothelial vessel with plasma) was quantified over time (Figure 5C) [17]. From these values, the velocity of fluorescence increase (the first derivative, dF/dt) was calculated using GraphPad Prism software (Figure 5D). The obtained first derivative was multiplied by a calibrator factor (Cf), yielding the thrombin concentration (Cf = nM/[dF/dt]) by using the H-transformation (Figure 5E) [22]. Altogether, adaptation of the calibrated automated thrombinography method by Hemker et al. [19,22] could be used to study thrombin generation inside the cancer-on-a-chip model.

### 3.4 Coculture of glioblastoma spheroids and HUVECs results in increased thrombin generation

**Figure 6:**
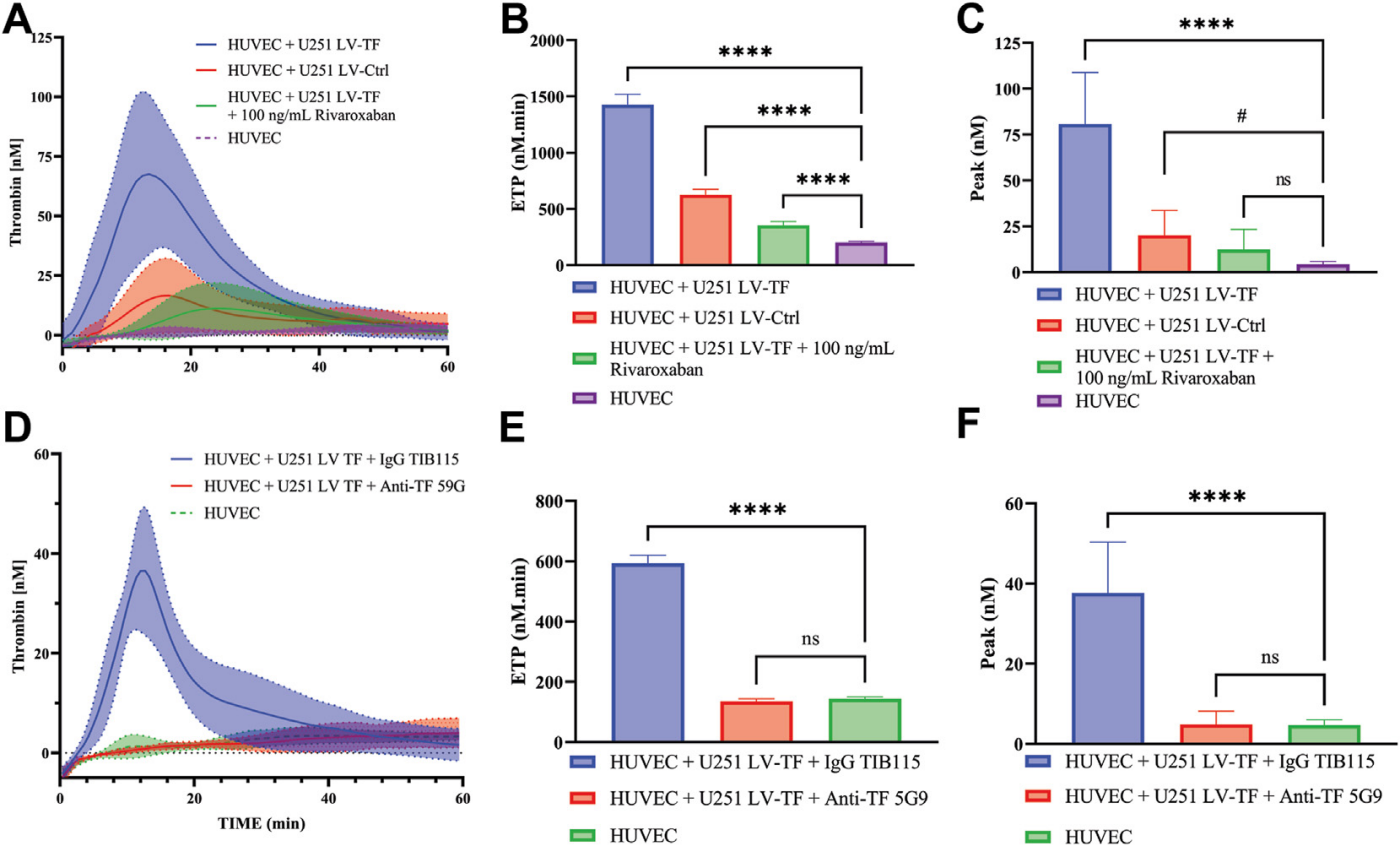
Thrombin generation promoted by U251 tumor spheroids is mediated by tissue factor (TF). (A, D) The coculture of human umbilical vein endothelial cell (HUVEC) vessels and U251 lentivirus (LV)-TF spheroids or U251 LV-control (Ctrl) spheroids was kept for 72 hours, and thrombin generation was measured inside the cancer-on-a-chip. Immunoglobulin (Ig)G TIB115 (50 μg/mL, IgG control), Ab TF-5G9 (50 μg/mL), and rivaroxaban (100 ng/mL) were incubated in plasma prior to the assay. The highlighted area represents the SD. (B, E) The endogenous thrombin potential (ETP) was calculated for each condition. (C, F) The highest thrombin peak from each chip was plotted in nanomolar. Data were pooled from 2 (D–F) and 3 independent experiments (A–C). Experiments were performed at least 3 times, and each condition was conducted in at least triplicate (*n* ≥ 3 chips per experiment). Statistical analysis was done using 1-way analysis of variance and Dunnett’s multiple comparison test. #*P* < .10. Ns, not significant.

To examine the procoagulant effects of glioblastoma cells, U251 LV-Ctrl and LV-TF spheroids were cocultured with HUVECs in the cancer-on-a-chip for 72 hours. Both spheroid types were able to increase thrombin generation in the chip compared with HUVECs alone (Figure 6A–C). The presence of U251 LV-TF spheroids resulted in increased thrombin generation in comparison with U251 LV-Ctrl spheroids, as visualized by a 7.0- and 3.1-fold increased endogenous thrombin potential (ETP), respectively, compared with thrombin generation by HUVECs alone (*P* < .0001 in both cases; Figure 6B). In line with this, the thrombin peak height increased from 4.29 ± 1.60 nM in HUVECs alone to 80.78 ± 28.18 nM (*P* < .0001) and 20.14 ± 13.57 nM (*P* < .09) upon coculture with U251 LV-TF or U251 LV-Ctrl spheroids, respectively (Figure 6C).

**Supplementary Figure 7:**
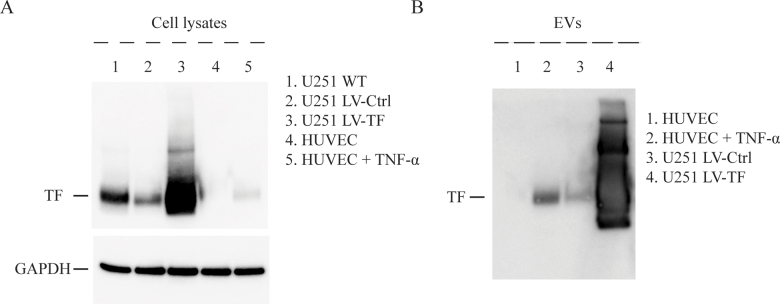


**Supplementary Figure 8:**
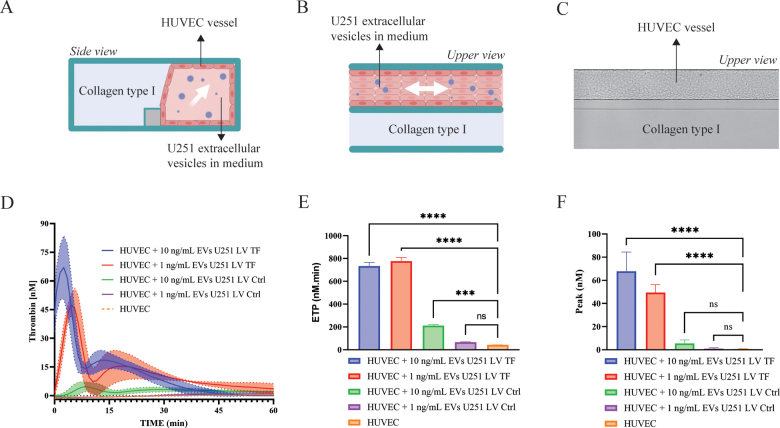


Thrombin generation inside the cancer-on-a-chip model presumably depends on secretion of TF-positive EVs by glioblastoma cells. Indeed, western blotting showed increased TF protein expression and secretion of TF-positive EVs by U251 LV-TF cells compared with U251 LV-Ctrl cells (Supplementary Figure S7). No TF expression was seen in HUVECs, but treatment with TNF-α did result in TF-positive cells and secretion of TF-positive EVs, which is in agreement with validation results of our adapted Hemker method (Figure 5). Additionally, thrombin generation was also performed in an OrganoPlate 2-lane (Supplementary Methods). EVs derived from U251 LV-Ctrl or U251 LV-TF were added to a single HUVEC vessel and incubated for 4 hours. Thrombin formation measured over time showed that there was a concentration-dependent increase in thrombin formation; ETP was increased at least 3.5-fold (*P* < .0001) after incubation of the vessels with 10 ng/mL of EVs derived from U251 LV-TF compared with EVs from U251 LV-Ctrl at the same concentration (Supplementary Figure S8).

**Supplementary Figure 9:**
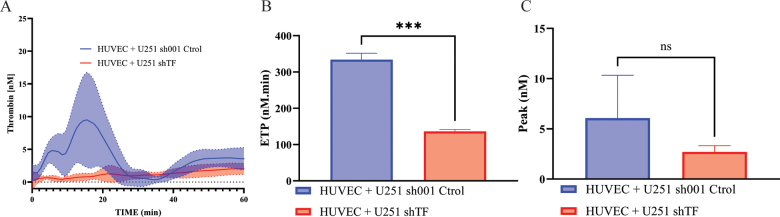


Finally, coculture of HUVECs together with spheroids with TF knockdown (U251 short hairpin TF) inside the cancer-on-a-chip showed reduced thrombin generation in comparison with Ctrl (U251 short hairpin Ctrl, Supplementary Figure S9), thus confirming a role for TF in glioblastoma-mediated coagulation.

### 3.5 Thrombin generation inside the cancer-on-a-chip is inhibited by anticoagulants

Finally, we inhibited different stages of thrombin formation using the coagulation inhibitors TF-5G9 (which blocks TF coagulant activity) and rivaroxaban (a direct oral anticoagulant that inhibits factor Xa and the prothrombinase complex). U251 LV-TF spheroids were used for this experiment due to their pronounced prothrombotic phenotype. In the presence of TF-5G9, the ETP was reduced by 4.4-fold (*P* < .0001; Figure 6D, E). This resulted in a thrombin peak similar to HUVECs alone (Figure 6F). Treatment with rivaroxaban also resulted in a 4.0-fold decrease of the ETP (*P* < .0001; Figure 6B), resulting in a 6.6-fold reduction of the thrombin peak (Figure 6C).

## 4 Discussion

In this study, we have developed a novel 3D cancer-on-a-chip model to investigate the dynamic interactions between cancer cells and the vasculature, providing an alternative to currently used animal models and 2D cell culture techniques. Traditional monolayer models have been the mainstay for *in vitro* thrombosis research. However, since the cancer-on-a-chip model relies on the grafting of a 3D spheroid in coculture with a HUVEC-based 3D vasculature, we believe that our model better represents cancer hypercoagulability than standard 2D approaches. Consequently, this model may contribute to the knowledge on the pathophysiological mechanisms behind CAT, which is of great importance for cancer patients with a high risk of VTE. Moreover, this model may be applicable to a large variety of (patho)physiological processes by providing great flexibility with regard to the type of materials and samples used.

Mice are the main animal model used for VTE studies. To induce VTE, it is necessary to perturb at least 1 of the 3 components of Virchow’s triad: blood stasis, vascular injury, and hypercoagulability. Well-known murine thrombosis models rely on endothelial injury induced by ferric chloride or rose bengal combined with laser irradiation. Another option is complete or partial blockage of the inferior vena cava through stasis (ligation) or stenosis models, respectively [6,25]. However, these models are invasive and rely on surgical procedures. A less invasive VTE model uses RNA interference to silence the genes encoding antithrombin and protein C in the liver, resulting in spontaneous generation of thrombi in the vasculature [26,27]. Still, this method is not ideal as thrombus formation does not occur at a predefined site.

In the context of CAT, thrombosis models need to be combined with orthotopic or subcutaneous tumor growth [28]. Therefore, immunodeficient mice have to be used, excluding a part of the immune system that may very well play a role in thrombus formation. In the current study, we present a cancer-on-a-chip model for studying cancer-related hypercoagulability, which provides promising benefits in comparison with the conventional approaches, consisting of components that are all human-derived, presenting a well-defined site of coagulation formation and allowing direct quantification of procoagulant activity. And importantly, this model reduces the need for animal experiments.

The vascular endothelium operates as a selective barrier between blood and underlying tissues. Vascular integrity is maintained by endothelial cell junctions consisting of adhesion molecules such as VE-cadherin and ICAM-1. In cancer, barrier maintenance is often impaired, resulting in leaky vessels and metastasis [29,30]. Especially in glioblastoma, vascular hyperpermeability is frequently observed [31], which is why this proof-of-principle study was performed with glioblastoma spheroids. Indeed, when coculturing glioblastoma spheroids together with vessel-forming HUVECs in the cancer-on-a-chip, the vessels showed increased ICAM-1 expression in the presence of both LV-Ctrl and LV-TF spheroids, whereas VE-cadherin expression was only increased in the U251 LV-TF condition (Figure 2). Furthermore, we observed a statistically significant increase in HUVEC permeability (Figure 2), demonstrating the influence of glioblastoma cells on the tumor microenvironment. This could be explained by glioblastoma-derived EVs expressing cytokines such as vascular endothelial growth factor, which were previously shown to enhance permeability and angiogenesis in human brain endothelial cells [32]. Indeed, U251 LV-TF cells showed increased secretion of TF-positive EVs, which resulted in increased thrombin generation—as confirmed by U251 spheroids with TF knockdown—and presumably affected endothelial integrity inside the cancer-on-a-chip.

In this study, we have used the calibrated automated thrombinography method developed by Hemker et al. [19] to quantify plasma-derived thrombin generation inside the chip (Figures 4 and 5). This method allows routine assessment of the clotting system in plasma, which helps clinicians to detect the risk of VTE or bleeding and determine optimal treatment strategies for each patient. Although normally applied in the absence of cells, the adapted method relies on the presence of endogenous TF within the cancer-on-a-chip model, probably expressed on glioblastoma-derived EVs or on HUVECs by cancer cell-mediated proinflammatory cytokines. This allowed us to directly measure the hypercoagulable effect of the glioblastoma spheroid on the vasculature within the cancer-on-a-chip, proving it as a convenient tool for studying cancer-induced procoagulant activity.

Thrombin generation in plasma inside the chip was enhanced in the presence of glioblastoma spheroids or tumor cell-derived EVs in comparison with HUVECs alone (Figure 6 and Supplementary Figure S8). These findings are in agreement with the increased VTE risk as seen in glioblastoma patients, in which TF is suggested to play an important role. This was confirmed by the use of anticoagulant modalities such as rivaroxaban and TF-5G9, which inhibited TF-mediated thrombin generation inside the vessels. As such, this model shows great potential for drug research and personalized medicine, which can be applied to a large variety of cancer types and patient material.

In this study, we present a novel 3D organ-on-a-chip model to investigate the molecular interactions between cancer cells and the vasculature that underlie CAT. Inside the cancer-on-a-chip, by combining HUVEC-based vessels with glioblastoma spheroids, we investigated the hypercoagulable effect of glioblastoma cells on endothelial permeability and thrombin generation, which may translate into VTE risk and metastasis within patients. Furthermore, this model could be used to test the effect of well-known anticoagulant modalities such as rivaroxaban, indicating a role in drug testing and personalized treatment. Altogether, the cancer-on-a-chip model may contribute to our knowledge on cancer-induced hypercoagulability and improve the prognosis of cancer patients who suffer from an increased risk of VTE, such as glioblastoma patients.

## Figures and Tables

**Supplementay Figure 10:**
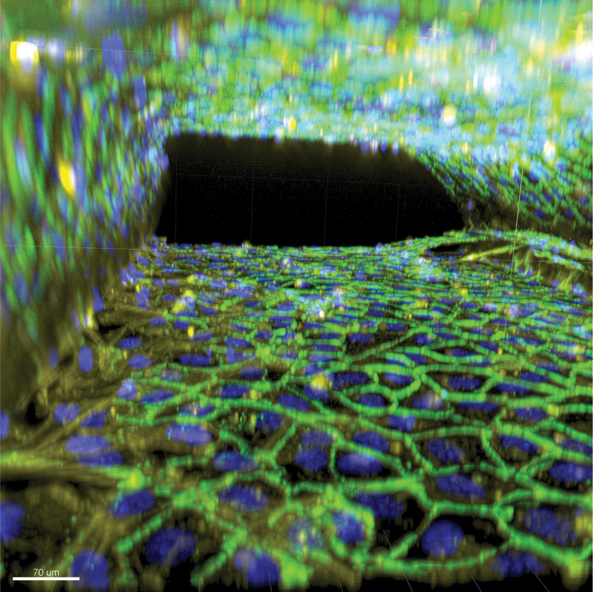


## References

[1] Mulder F.I., Horváth-Puhó E., van Es N., van Laarhoven H.W.M., Pedersen L., Moik F. (2021). Venous thromboembolism in cancer patients: a population-based cohort study. Blood.

[2] Timp J.F., Braekkan S.K., Versteeg H.H., Cannegieter S.C. (2013). Epidemiology of cancer-associated venous thrombosis. Blood.

[3] Chew H.K., Wun T., Harvey D., Zhou H., White R.H. (2006). Incidence of venous thromboembolism and its effect on survival among patients with common cancers. Arch Intern Med.

[4] Khorana A.A., Francis C.W., Culakova E., Kuderer N.M., Lyman G.H. (2007). Thromboembolism is a leading cause of death in cancer patients receiving outpatient chemotherapy. J Thromb Haemost.

[5] Rondon A.M.R., Kroone C., Kapteijn M.Y., Versteeg H.H., Buijs J.T. (2019). Role of tissue factor in tumor progression and cancer-associated thrombosis. Semin Thromb Hemost.

[6] Albadawi H., Witting A.A., Pershad Y., Wallace A., Fleck A.R., Hoang P. (2017). Animal models of venous thrombosis. Cardiovasc Diagn Ther.

[7] Yust-Katz S., Mandel J.J., Wu J., Yuan Y., Webre C., Pawar T.A., Armstrong (2015). Venous thromboembolism (VTE) and glioblastoma. J Neurooncol.

[8] Kaptein F.H.J., Stals M.A.M., Kapteijn M.Y., Cannegieter S.C., Dirven L., van Duinen S.G. (2022). Incidence and determinants of thrombotic and bleeding complications in patients with glioblastoma. J Thromb Haemost.

[9] Koshy M., Villano J.L., Dolecek T.A., Howard A., Mahmood U., Chmura S.J. (2012). Improved survival time trends for glioblastoma using the SEER 17 population-based registries. J Neurooncol.

[10] Lyman G.H., Bohlke K., Falanga A., American Society of Clinical Oncology (2015). Venous thromboembolism prophylaxis and treatment in patients with cancer: American Society of Clinical Oncology clinical practice guideline update. J Oncol Pract.

[11] D'Asti E., Magnus N., Meehan B., Garnier D., Rak J. (2014). Genetic basis of thrombosis in cancer. Semin Thromb Hemost.

[12] Kapteijn M.Y., Lanting V.R., Kaptein F.H.J., Guman N.A.M., Laghmani E.H., Kuipers T.B. (2023). RNA-sequencing to discover genes and signaling pathways associated with venous thromboembolism in glioblastoma patients: a case-control study. Thromb Res.

[13] van Duinen V., Stam W., Borgdorff V., Reijerkerk A., Orlova V., Vulto P. (2019). Standardized and scalable assay to study perfused 3D angiogenic sprouting of iPSC-derived endothelial cells *in vitro*. J Vis Exp.

[14] Vreeken D., Bruikman C.S., Cox S.M.L., Zhang H., Lalai R., Koudijs A. (2020). EPH receptor B2 stimulates human monocyte adhesion and migration independently of its EphrinB ligands. J Leukoc Biol.

[15] Rondon A.M.R., de Almeida V.H., Gomes T., Verçoza B.R.F., Carvalho R.S., König S. (2018). Tissue factor mediates microvesicles shedding from MDA-MB-231 breast cancer cells. Biochem Biophys Res Commun.

[16] Kapteijn M.Y., Zwaan S., Ter Linden E., Laghmani E.H., van den Akker R.F.P., Rondon A.M.R. (2023). Temozolomide and lomustine induce tissue factor expression and procoagulant activity in glioblastoma cells *in vitro*. Cancers (Basel).

[17] Schindelin J., Arganda-Carreras I., Frise E., Kaynig V., Longair M., Pietzsch T. (2012). Fiji: an open-source platform for biological-image analysis. Nat Methods.

[18] van Duinen V., van den Heuvel A., Trietsch S.J., Lanz H.L., van Gils J.M., van Zonneveld A.J. (2017). 96 perfusable blood vessels to study vascular permeability *in vitro*. Sci Rep.

[19] Hemker H.C., Giesen P., Al Dieri R., Regnault V., de Smedt E., Wagenvoord R. (2003). Calibrated automated thrombin generation measurement in clotting plasma. Pathophysiol Haemost Thromb.

[20] Laan S.N.J., Dirven R.J., Bürgisser P.E., Eikenboom J., Bierings R. (2023). SYMPHONY consortium. Automated segmentation and quantitative analysis of organelle morphology, localization and content using CellProfiler. PLoS One.

[21] Postma R.J., Broekhoven A.G.C., Verspaget H.W., de Boer H., Hankemeier T., Coenraad M.J. (2024). Novel morphological profiling assay connects *ex vivo* endothelial cell responses to disease severity in liver cirrhosis. Gastro Hep Adv.

[22] Hemker H.C., Kremers R. (2013). Data management in thrombin generation. Thromb Res.

[23] Duarte R.C.F., Ferreira C.N., Rios D.R.A., Reis H.J.D., Carvalho M.D.G. (2017). Thrombin generation assays for global evaluation of the hemostatic system: perspectives and limitations. Rev Bras Hematol Hemoter.

[24] Chen Y., Zheng Y., Xin L., Zhong S., Liu A., Lai W. (2019). 15-epi-lipoxin A(4) inhibits TNF-α-induced tissue factor expression via the PI3K/AKT/ NF-κB axis in human umbilical vein endothelial cells. Biomed Pharmacother.

[25] Diaz J.A., Saha P., Cooley B., Palmer O.R., Grover S.P., Mackman N. (2019). Choosing a mouse model of venous thrombosis. Arterioscler Thromb Vasc Biol.

[26] Safdar H., Cheung K.L., Salvatori D., Versteeg H.H., Laghmani el H., Wagenaar G.T. (2013). Acute and severe coagulopathy in adult mice following silencing of hepatic antithrombin and protein C production. Blood.

[27] Heestermans M., Salloum-Asfar S., Streef T., Laghmani E.H., Salvatori D., Luken B.M. (2019). Mouse venous thrombosis upon silencing of anticoagulants depends on tissue factor and platelets, not FXII or neutrophils. Blood.

[28] Hisada Y., Mackman N. (2018). Mouse models of cancer-associated thrombosis. Thromb Res.

[29] Nikitenko L.L. (2009). Vascular endothelium in cancer. Cell Tissue Res.

[30] Claesson-Welsh L., Dejana E., McDonald D.M. (2021). Permeability of the endothelial barrier: identifying and reconciling controversies. Trends Mol Med.

[31] Dubois L.G., Campanati L., Righy C., D'Andrea-Meira I., Spohr T.C., Porto-Carreiro I. (2014). Gliomas and the vascular fragility of the blood brain barrier. Front Cell Neurosci.

[32] Treps L., Perret R., Edmond S., Ricard D., Gavard J. (2017). Glioblastoma stem-like cells secrete the pro-angiogenic VEGF-A factor in extracellular vesicles. J Extracell Vesicles.

