# Supplementary material for: Modeling cancer-associated hypercoagulability using glioblastoma spheroids in microfluidic chips
Source: Res Pract Thromb Haemost. 2024 Jun 17;8(5):102475. doi: 10.1016/j.rpth.2024.102475 (PMC11391032; doi:10.1016/j.rpth.2024.102475)
Supplement: Supplementary Methods [file mmc2.docx]

**Supplementary Materials**

**Modeling cancer-associated hypercoagulability using glioblastoma spheroids in microfluidic chips**

**Authors:** Maaike Y. **Kapteijn**^1^, Monika **Yanovska**^1^, El Houari **Laghmani**^1^, Rudmer J. **Postma**^2^, Vincent **van** **Duinen**^2,3^, Betül **Ünlü**^1^, Karla **Queiroz**^3^, Anton Jan **van Zonneveld**^2^, Henri H. **Versteeg**^1^ and Araci M. R. **Rondon**^1,*^

^1^Division of Thrombosis and Hemostasis, Einthoven Laboratory for Vascular and Regenerative Medicine, Leiden University Medical Center, Leiden, The Netherlands; ^2^Division of Nephrology, Einthoven Laboratory for Vascular and Regenerative Medicine, Leiden University Medical Center, Leiden, The Netherlands; and ^3^Mimetas BV, Leiden, The Netherlands.

**Corresponding author:** Dr. Araci Rondon, Einthoven Laboratory for Vascular and Regenerative Medicine, Div. of Thrombosis and Hemostasis, Department of Internal Medicine. Leiden University Medical Center. Albinusdreef 2, C7-Q, 2333 ZA Leiden, The Netherlands. Phone number: +31715268150. Email: [a.m.da_rocha_rondon@lumc.nl](mailto:a.m.da_rocha_rondon@lumc.nl)

**Supplementary**

**Supplementary** **Materials and methods**

Immunostaining quantification

The area of fluorescence intensity of VE-cadherin was determined by applying a threshold, followed by quantification of the area using FIJI [1], (**Supplementary Figure 3**). Single-cell ICAM-1 intensities were quantified using R (version 4.1.1) [2] and the R-package EBImage (version 4.29.2) [3], based on [4]. Individual nuclei were identified from Hoechst-channel images by first applying a local threshold, followed by watershed segmentation to identify individual nuclei. Debris was filtered out by applying a lower threshold for nuclei size. Cells were identified by Voronoi-Based segmentation using the phalloidin signal to identify cell borders, using individual nuclei as seeds. To filter out larger debris structures, an upper threshold was applied for the nuclei size. Cell-segmentations containing these large debris structures were discarded. R-package EBImage “compute” functions were used to compute the intensity of ICAM-1 on a single cell level (**Supplementary Figure 5)**. ICAM-1 positive cells were identified and counted by plotting ICAM-1 mean intensities in a density plot followed by identifying the intensity peak of positive cells and calculating the percentage of cells above this cutoff.

Weibel-Palade bodies (WPB) were quantified as described before [5], (**Supplementary Figure 4)**. In short, CellProfiler (Version 4, Broad Institute) was used to identify nuclei from Hoechst-channel images. Cells were identified from VE-Cadherin images by Voronoi-Based segmentation, using individual nuclei as seeds. Weibel-Palade bodies were identified from the vWF channel images by global thresholding, determining positive signal by Otsu thresholding. WPBs were assigned to each cell by distance measure to the cell body.

Microfluidic culture OrganoPlate 2-lane

To seed the OrganoPlate 2-lane (9605-400-B, Mimetas, The Netherlands) chips, 2 µL of collagen mix was added into the gel inlet of each chip and incubated for 15 minutes at 37°C and 5% CO_2_. Following this, 2 µL of 1x10^7^ HUVECs/mL was added to the medium inlet, followed by 50 µL of EGM2 and incubated for 2 hours at 37°C and 5% CO2. Afterward, 50 µL of EGM2 was added to the medium outlet. The plate was then positioned on an interval rocker platform at 7 degrees inclination with 8 minutes cycle time to allow continuous bidirectional flow for at least 4 days.

Thrombin generation inside the cancer-on-a-chip model using extracellular vesicles

Extracellular vesicles were diluted in EGM2 to concentrations of 1 and 10 ng/mL, and then added to chips for four hours. The chips were washed two times with 50 µL of pre-warmed HBSS-. Next, 80 µL of 1:1 plasma in HBSS- containing 50 µg/mL CTI was added per chip. Thrombin formation was initiated by adding 10 µL of pre-warmed fluorescence-substrate buffer containing calcium chloride. Chips containing thrombin calibrator were used, following the same protocol described for the OrganoPlate Graft. The fluorescence emitted was measured over time in the HUVEC vessel (**Supplementary Figure 8**).

***Supplementary Figures*** ***Legends***

***Supplementary Figure 1. U251 spheroid formation.****Different concentrations (2,500, 5,000, 10,000 cells) of U251 LV-Ctrl or U251 LV-TF cells were cultured in round bottom low attachment 96 wells plates (Corning, USA) in 200 µL of DMEM with 10% FBS and 1% P/S per well for 4 days to generate spheroids. Representative pictures of the spheroids at different time points are shown in (A). Spheroid diameter was measured over time using FIJI (B, 4 spheroids per condition). The highlighted area represents the SD. Bar corresponds to 500 µm. # represents p<0.10.*

**Supplementary Figure 2.** Immunofluorescence images of the middle section of a HUVEC-vessel inside a cancer-on-a-chip, created with Imaris (Oxford Instruments, UK). A-B) Lateral view. C) Longitudinal view of a vessel. Blue: DNA/Hoechst 33342; green: VE-cadherin; yellow: phalloidin. Bar: 70 µm

***Supplementary Figure 3. Example of quantification of VE-cadherin area per cell.*** *Cells were stained with Mouse anti-human VE-cadherin, followed by Goat anti-mouse IgG Alexa 488 and Hoechst 33342. A) A threshold was applied to the nuclei images and the number of nuclei was counted using FIJI. B) A threshold was applied to VE-cadherin images and the area was measured using FIJI.*

***Supplementary Figure 4. Example of quantification of Weibel-Palade bodies (WPB) per cell.*** *A) To identify WPB, fixed cells were incubated with Rabbit anti-human vWF, followed by Goat anti-rabbit IgG Alexa 647 counterstaining. B) WPB were segmented and counted in CellProfiler by first identifying single cells using nuclei as seeds and region propagation to identify cells. WPB were identified by global thresholding using the Otsu threshold as cutoff, according to* *[5].*

***Supplementary Figure 5. Example of quantification of the intensity of ICAM staining.*** *A) Cells were stained with Mouse anti-human ICAM-1, followed by Goat anti-mouse IgG Alexa 488. B) Single cells were identified by first using the Hoechst 33342 signal to identify nuclei, followed by region propagation by the phalloidin signal to identify cell borders, as described in Supplementary Materials and Methods. C) Mean intensity of the ICAM-1 signal was computed on a single cell level. Results of each single cell mean intensity was plotted in a density plot. Subsequently, the ICAM-1 positive cell population was identified from the HUVEC only condition. This cutoff was used to calculate the positive populations for each condition.*

*Supplementary Figure 6. Thrombin generation inside the cancer-on-a-chip based on fluorescence intensity using the FluCa-kit for 60 min. A) HUVECs only and B) HUVECs co-cultured with a U251 LV-TF spheroid for 4 days. 80 μL of blood plasma was added after washing the chips twice with HBSS without calcium and magnesium. To start the reaction, 20 μL/chip of Fluo-buffer + Fluo-substrate (containing calcium chloride) was added, and the fluorescence intensity was measured over time using FIJI.*

**Supplementary Figure 7**. A) Western blotting was performed to determine TF protein expression using TF-antibody 10H10 and GAPDH as housekeeping protein in cells. HUVECs were stimulated for 4h with 10 ng/mL TNF-α. B) Extracellular vesicles (EVs) derived from different cell types were purified by centrifugation and Western blotting was performed to measure TF protein expression on EVs. The same concentration of cell lysate or EV lysate was added for all conditions. WT: wild type.

***Supplementary Figure 8. Cancer-associated hypercoagulability-on-a-chip using extracellular vesicles (EVs) from U251 cells.*** *Schematic side view (A) and upper view (B) representation of one OrganoPlate 2-lane chip. HUVEC vessels were formed in direct contact with Collagen type I and kept in culture for at least 4 days. C) Live image of a HUVEC vessel. D) After stimulating HUVEC vessels with EVs for 4 hours, thrombin generation was performed inside the vessels. The endogenous thrombin potential (E) and highest thrombin peak (F) were calculated. Graphics are representatives of one experiment. Experiments were performed at least two times, and each condition was conducted at least in quadruplicate.*

***Supplementary Figure 9.*** *A) Spheroids from U251 cells with TF knock down showed an apparent reduction in thrombin formation after 4 days of co-culture with HUVECs in the cancer-on-a-chip model. B) The endogenous thrombin potential (ETP) was calculated. C) The highest thrombin peak from each chip was plotted in nanomolar. Representative data of two experiments (n≥3).*

***Supplementary Video 1****. Immunofluorescence video of the middle section of a cancer-on-a-chip, created with Imaris. Blue: DNA/Hoechst 33342; green: VE-cadherin; yellow: phalloidin.*

**Supplementary** **References**

[1] V. van Duinen, A. van den Heuvel, S.J. Trietsch, H.L. Lanz, J.M. van Gils, A.J. van Zonneveld, P. Vulto, T. Hankemeier, 96 perfusable blood vessels to study vascular permeability in vitro, Sci Rep 7(1) (2017) 18071.

[2] R.C. Team, R: A Language and Environment for Statistical Computing, R Foundation for Statistical Computing, 2021.

[3] G. Pau, F. Fuchs, O. Sklyar, M. Boutros, W. Huber, EBImage--an R package for image processing with applications to cellular phenotypes, Bioinformatics 26(7) (2010) 979-81.

[4] R.J. Postma, A.G.C. Broekhoven, H.W. Verspaget, H.d. Boer, T. Hankemeier, M.J. Coenraad, V.v. Duinen, A.J.v. Zonneveld, Novel Morphological Profiling Assay Connects ex Vivo Endothelial Cell Responses to Disease Severity in Liver Cirrhosis, Gastro Hep Advances 3 (2024) 238–249.

[5] S.N.J. Laan, R.J. Dirven, P.E. Bürgisser, J. Eikenboom, R. Bierings, Automated segmentation and quantitative analysis of organelle morphology, localization and content using CellProfiler, PLoS One 18(6) (2023) e0278009.
